# 

**Published:** 1890-08-16

**Authors:** 


### General Charities.

[This Column is devoted to an account of work of charitable institutions other than Medical Charities. The Editor will be glad to receive reports or news of work at 140, Strand. *Philanthropy* should be plainly written on the envelope.]

The names of those who are pleading for help to give **ONE DAY HOLIDAYS TO POOR CHILDREN** are legion; want of space, we regret to say, prevents our printing them. We can so readily sympathise with the organizers and believe what a pleasure this one day gives, but at the same time, we cannot agree with those who urge that the necessity for "the Day in the Country" exists because it is impossible to send all the London children away for a more lengthened period. Is it impossible? We doubt it very much. London is a very rich and a very generous city. What we do say most emphatically is, that it will never be done while there are the hundred and one promoters of different fresh air schemes, and while in some cases total lack of organization exists. The "one day" is a sort of compensation to those who are not lucky enough to get away and "stay in the country," at least that is what it ought to be. In New York nearly all the children go now. We in London ought soon to be in the same position. It is very well to laugh at "Organization, and say we prefer "Charity," but then organization means trouble, and it is easy to be lazy in our charitable endeavours and to give indiscriminately and unworthily. There are many districts in London which have nobody expending their efforts on the children's welfare; there are others where three or four different people are working three or four different schemes, which of necessity clash. Then, again, some of the small schemes for "holidays" give all their tickets free, shower them down on people who are perfectly well able to contribute something towards their children's enjoyments, and thereby allow other children a chance. Not only this, but cases have occurred where these indiscriminate givers have so overlapped each other's efforts that one child has had three holidays in a year, and another child none at all. Other cases come to our knowledge where the heads of Sunday-schools or day-schools of various sects give a free holiday to their pupils in order to induce them to attend their particular school. They in fact are using the holiday only to proselytize. What trouble would it be to people, before commencing their efforts in a parish, to find out whether others are already working there; if so, either co-operate or take their time, trouble, and money to some other district. And for the rest, we earnestly ask those who are thinking of starting new schemes to aid the already-established ones. Our sympathies are honestly with all who try to help without pauperizing in aiding the phys'cal, mental, and moral development of the children in the city, but we urge our readers to remember that co-operation is strength, that many separate undertakings mean waste of money as well as interest, and that to succeed we must unite in one great effort for one great good, and we must get persons of every shade of opinion in all conditions of life to join hands in the struggle to alleviate the sorrows and aid the joys of little children. Can there be greater charity than this?

Thirteen hundred fountains and troughs are kept in order in London and its suburbs by the **METROPOLITAN DRINKING FOUNTAIN ASSOCIATION**, the cost for which alone during the past year has exceeded £1,500. Subscriptions are much required, and the amount of physical suffering this association saves men and beasts ought to be sufficient commendation of it. Secretary, M. W. Milton, Esq. Offices, 11, Victoria Street, S.W.

A society, which from its earliest days has gone quietly and unobtrusively on its way, doing much good work ever since 1859, is the **SOCIETY FOR THE EMPLOYMENT OF WOMEN.** It does its helping in a way which appeals to us. Money is never given, the society aiming at maintaining the self-respect of women by enabling them to help themselves. Apprentice fees are often paid for girls. The City and Guilds of London Technical Institute have this year put by £100 to be spent through the agency of this society for paying apprentice fees. Besides what the society does for the training of girls, it has a register for educated women who seek employment at 22, Berners Street. Governesses, accountants, type-writers, needlewomen, decorative artists, compositors, may all be found on applying to the office, and what is still more, none are registered who are not found to be thoroughly competent in the work they profess to do. Technical education is now one of the cries of the day, but when this society started it struggled against indifference to special thorough training. Its efforts are much crippled through want of adequate funds, and we recommend any generous-minded person willing to assist to bear in mind the name of the secretary, Miss King, 22, Berners Street, Oxford Street.

### NOTICE TO CORRESPONDENTS.

All MS., Letters, Books for Review, and other matters intended for the Editor should be addressed The Editor, The Lodge, Porchester Square, London, W.

The Editor cannot undertake to return rejected M.S., even when accompanied by stamped directed envelope.

**Notice to Advertisers and Subscribers.**

All Advertisements, Orders for Copies of The Hospital, and Business Communications should be addressed to The Publisher, *not to the Editor*, The Hospital, 140, Strand, W.C.

**Bound Volumes of "The Hospital."**

Vol. VI., containing full page portraits of T.R.H. the Prince and Princess of Wales, and Miss Florence Nightingale, is obtainable at 4s., post free.

Orders will be received for Vol. VII., 4s. post free. Cases for binding may be had of the Publisher, at 1s. 6d. each.

**To Residents, Secretaries, and Matrons.**

The Editor will be glad to have the Regulations and each Report as issued by Asylums, Hospitals, Convalescent and Nursing Institutions, as well as those of other Charities, and an early copy in duplicate of all Appeals and Festival Notices, etc., addressed to *The Editor, The Lodge, Porchester Square, London, W.* Any superintendent, secretary, matron, or other chief official who regularly sends such information may be registered, on application to the Publisher, to receive free of cost a cover for binding The Hospital in half-yearly volumes.

### BURDETT'S HOSPITAL ANNUAL FOR 1890

Is now ready, and may be obtained of the Publisher, The Hospital Offices, 140, Strand, W.C., price 2s. 6d. limp boards, or 3s. 6d. cloth. All orders must be accompanied by a remittance.

### STUDENTS' COLUMN.

### UNIVERSITY OF EDINBURGH.

### FIRST PROFESSIONAL PASSES.

The following is the official list of candidates who passed the first professional examination in July :—

Three Subjects.—S. J. Aarons, C. H. Adamson, George Anderson, William Anderson, W. C. Anderson, M.A., A. W. M. Auden, B.A., M. A. Bamby, W. J. Bell, Robert Bennie, J. G. Bentham, S. F. Blakely, J. E. Blackburn, R. W. Blair, R. St. G. S. Bond, C. W. Branch, D. J. S. Burt, James Cappie, William Catto, M.A., R. M. Clark, W. R. Center (with distinction), C. M. Christy, R. A. G. Constantian, Charles Crerar, Donald Crerar, J. R. Crease, Andrew Croll, Ignatius Crook, N. C. Das, A. N. Davies, W. J. N. Davis, E. P. Dickin, G. A. Dickson, E. R. Dodds, J. D. Doherty, E. C. Edwards, L. C. Edwards, J. A. Featherstone, P. Fick, J. S. Flett, M.A., T. D. Forbes, Harvie Forrester, Arthur Foster, P. W. Freyer, J. A. Fullarton, Thomas Gibson, M.A., H. H. Gill, Y. Goldie, Douglas Gordon, M. W. Gouhy-Chion, Lachlan Grant, W. Grant, James Gray, W. A. Gray, B.A., Arthur Gwyther, H. H. Hadden, F. H. Hardman, A. G. Hayden, George Hodges, C. A. Hogg, L. J. Howes, C. J. Hugo, J. A. Hunter, E. W. J. Ireland, W. E. Jameson, E. M. Jennings, S. E. Johnston, C. B. Johnstone, D. A. Johnstone, A. K. Jones, C. W. A. Jones, J. H. Jones, W. H. Jones, Charles Kerr, David Lawson, M.A., J. A. Lee, R. E. Legat, G. F. Leicester, J. G. Leslie, Robert M'Camon, A. W. J. MacFadden, William Mackirdy, Andrew M'Neil, B.A., Archibald Macnicol, R. H. Makgill, W. C. Marsdin, W. R. Martine, A. C. Matchett, J. M. Menzies, T. B. Moore, B.A., R. C. Morris, M.A., A. J. M. Paget, J. B. Pearson, Tom Pettey, Andrew Philip, George Phillips, A. M. N. Pringle, S. W. Prowse, B.A., Peter Rattray, C. H. Ridley, Thomas Roberts, E. C. Robertson, Alexander Rodger, A. P. Ross, G. H. Ruston-Harrison, J. M. Rutherford, C. R. Sheward, G. S. Shirt, John Sorley, M.A., David Steele, J. G. Standing, A. P. Steavenson, F. W. M. Stephenson, W. F. Stevenson, W. R. Strapp, H. S. Sturrock, D. W. Sutherland, John Tennant, M.A., David Thomas, M.A., C. G. Thompson, W. H. Thomson J. B. Tough, W. B. Turnbull, A. C. Turner, H. D. Vallant, Frederick Wilson, G. H. Wilson, J. J. Wilson, J. T. Wright.

### SECOND PROFESSIONAL PASSES.

The following is the official list of students who passed in the second professional examination this month:—

A. J. Anderson, James Anderson (M.A.), F. R. K. Ball, J. T. Balmford, L. G. Barbeau, G. F. Barnardo, F. C. Bell, A. F. M. Berkeley, George Bill, J. J. Brennan, Alexander Brownlie (with distinction), S. C. Brush, F. S. Butler, A. N. S. Carmichael, C. W. Chapman, H. A. Clark, J. C. Coker, J. W. Compton, R. A. Corson, J. F. Crombie, H. W. Crosse, J. W. Dawson, E. C. Dobie, R. H. Drennan, Alfred Duke (M.A.); C. C. Easterbrook, M.A. (with distinction); Samuel Edgerley (M.A.), A. M. Elliott, A. C. Elliott, Michael Emin, J. L. Fenton (B.A.), William Fletcher, W. R. Forbes, William Forsyth, J. R. Foster, Charles Frier, William Gordon, W. H. Gregory, W. C. Grosvenor (M.A.), F. E. Gunter, T. C. Guthrie, A. S. Harriss, A. H. Hopkins, E. C. W. Hughes-Games, R. H. Humphreys, Alexander Ingrim, C. H. Isard, P. P. H. Jagannadham, B.A.; R. J. Johnston (with distinction); George Johnston, H. B. Jones, H. B. Knox, Robert Knox, R. M. Leslie, M.A., B.Sc. (with distinction), J. R. Liddell, E. S. Littlejohn, Donald Macaulay, M.A. (with distinction); Alexander M'Ewen, Robert Macfarlane, T. N. Macgowan, F. W. Mackay, A. J. Mackintosh, Duncan M'Neill, H. B. Mapleton (B.A.), Charles Martin, T. M. Martin, C. E. Maude (B.A.), J. S. Maynard, A. J. Meikle, James Miller, P. St. C. More, D. S. Morrison (M.A.), G. C. Munnik, C. R. Nair, E. M. Nicholl, John Nicholson, Alexander Van Niekerk, Samuel Oddie, G. G. Osborn, T. L. Parry, D. W. T. Paterson, C. R. Pearce, William Peart-Thomas, R. O. Petrie (M.A.), James Philip, F. L. Pochin, J. C. Potter, G. H. Prance, W. G. Putnam, Stanley Riseley, Allan Robertson, J. B. Scott, R. W. L Scott, Ratou Scheult, C. D. Simitopoulos, H. J. Slane, A. S. Smith, J. G. Smith, P. C. C. Smith, W. J. J. Stewart, T. N. Stuart, C. A. Sturrock (M.A.), James Sutherland, John Sutherland, W. S. Syme, George Templeton, Bernard Thomas, J. T. Thyne, Walter Tibbits, H. S. Walker, T. D. Walker, W. J. Walker, B.A. (with distinction), R. B. Wallace, J. M. Walls, A. E. Watson, E. J. Weightman, Victor Werdmüller, Harry Whittome, J. D. Williams, R. M. Williams, G. R. Wilson, W. H. Wilson.

### UNIVERSITY OF LONDON.

### INTERMEDIATE EXAMINATION IN MEDICINE, JULY, 1890.

### EXAMINATION FOR HONOURS :—

ANATOMY.—*First Class*. Carwardine, Thomas (Exhibition and Gold Medal), Middlesex Hospital; Piercy, Ann Frances (Gold Medal). London School of Medicine for Women. *Second Class*: *Randall, Martin, B.A., University College; *Rogers, Leonard, St. Mary's Hospital; Wesley, Frank William, University College; James, Thomas Llewelyn, Yorkshire College. *Third Class*: *Jones, Lillie Mabel Agnes, London School of Medicine for Women; *Pantin, Charles Satchell, Guy's Hospital; *Spencer, Charles George, University College.

PHYSIOLOGY AND HISTOLOGY.—*First Class*: *Piercy, Ann Frances, London School of Medicine for Women; *Selby, Edmond Wallace, University College; *Spencer, Charles George, University College. *Second Class*: *Carwardine, Thomas, Middlesex Hospital; *Rogers, Leonard, St. Mary's Hospital; *Wesley, Frank William, University College; Jaffé, Charles Samson, St. Thomas's Hospital; Chadburn, Maud Mary, London School of Medicine for Women. *Third Class*: Lane, Clayton Arbuthnot, St. Mary's Hospital; *Pantin, Charles Satchell, Guy's Hospital; *Parsons, Christopher Thackray, St. Mary's Hospital; *Sharp, Arthur John, Guy's Hospital; +Edgcombe, Wilfrid, University College, Liverpool; †Lucas, William Freer, Middlesex Hospital; Iredale, Joseph Leonard, Leeds School of Medicine.

ORGANIC CHEMISTRY.—*First Class*: Hopkins, Frederick Gowland (Exhibition and Gold Medal), Guy's Hospital. *Second Class*: Winston, William Bamford, St. Thomas's Hospital.

MATERIA MEDICA AND PHAMACEUTICAL CHEMISTRY.—*First Class*: Piercy, Ann Frances (Exhibition and Gold Medal), London School of Medicine for Women; * Selby, Edmond Wallace, University College; * Stephens, George Arbour, B.Sc., University College; Randall, Martin, University College; Baker, John Charles, B.A., St. Bartholomew's Hospital; Edgecombe, Wilfrid. University College, Liverpool. *Second Class*: Jones, Arthur Bassett, University College; Sharp, Arthur John, Guy's Hospital; Rogers, Leonard, St. Mary's Hospital; Parsons, Christopher Thackray, St. Mary's Hospital. *Third Class*: Jaffé, Charles Samson, St. Thomas's Hospital; Winston, William Bamford, St. Thomas's Hospital; Hopkins, Frederick Gowland, Guy's Hospital.

### PASS LIST.

ENTIRE EXAMINATION.—*First Division*: Ballance, Hamilton Ashley, University College; Charles, Blackwell, St. Bartholomew's Hospital; Fletcher, John Howard, Owens College: Fuller, Charles Arthur, St. Mary's Hospital; Grant, Francis Octavius, St. Thomas's Hospital; Griffiths, John Howell, St. Bartholomew's Hospital; Grinling, Frederick Newman, University College; Gully, Robert Cullum, St. Bartholomew's Hospital; Hainworth, Edward Marrack, St. Thomas's Hospital; Harvey, John Owen, St. Bartholomew's Hospital; Heather, Benjamin Grant, St. Thomas's Hospital; Martin, Arthur James, Queen's College, Birmingham; Morton, John Leyden, St. Mary's Hospital; Mostertz, Julius, University College; Paine, Alexander, St. Mary's Hospital; Perram, Charles Herbert, St. Bartholomew's Hospital; Smith, Edwin, St. Thomas's Hospital; Turner, William, King's College; Ward, Charles Frederick Myers, Queen's College, Birmingham; Whitaker, James Smith, Owens College and Royal Infirmary, Manchester. *Second Division*: Breeze, Gabrielle Ruth S., London School of Medicine for Women; Coleman, Francis Jordan, Guy's Hospital; Crowley, Ralph Henry, St. Bartholomew's Hospital; Dukes, Edmund Sprague, Guy's Hospital; Harrison, Charles Joseph, University College; Heath, Arthur Douglas, University College; Hicks, Thomas William, St. Thomas's Hospital; Jäger, Harold Julius, King's College; Johnson, William John, Guy's Hospital; Jones, John, University College; Levy, Alfred Goodman, University College; Nesfield, Stephen, Owens and University Colleges; Pace, Harold Everett, London Hospital; Redpath, William, St. Thomas's Hospital; Sawers, John Lorimer, University College; Smith, Geo. Samuel Stone, St. Thomas's Hospital; Soden, Wilfred Newell, St. Bartholomew's Hospital; Stokes, Whitley, St. Thomas's Hospital; Ta'Bois, Alfred Charles, St. Bartholomew's Hospital; Thomas, William Martyn, Charing Cross Hospital; Vincent, Thomas Swale, Queen's College, Birmingham; Wells, Sydney Russell, *B.Sc.*, St. George's Hospital.

EXCLUDING PHYSIOLOGY.—*First Division*: Bathurst, Lullum Wood, St. Bartholomew's Hospital; Thomas, William, Yorkshire College. *Second Division*: Bakewell, Robert Turle, University College; Card, Alfred Herbert, Guy's Hospital; Hazell, Frederick, Guy's Hospital; Jewell, William Henry, Guy's Hospital; Jones, Alfred George, Middlesex Hospital; Martyn, George, King's College; Park, Wm. Chas. Cunningham, Guy's Hospital; Rawlings, John Dunnell, St. Bartholomew's Hospital; Smith, Reginald, Owens College.

PHYSIOLOGY ONLY.—*First Division*: Atwool, William Tillinghast, St. Mary's Hospital; Fraser-Luckie, Henry O'N., St. Bartholomew's Hospital; Parker, Henry Thomas, St. Bartholomew's Hospital; Pickering, Rowland N. U., St. Bartholomew's Hospital; Rogerson, Clement Michael, Yorkshire College; Stephens, Richard John, King's College; Tildesley, Josiah Percival, Queen's College, Birmingham; Tracey, Henry Eugene, St. Bartholomew's Hospital; Tebb, Albert Edward, Guy's Hospital. *Second Division*: Thorne, Atwood, St. Mary's Hospital.

N.B.—The names of Candidates who have obtained Honours do not appear in the Pass List.

* † Denotes equality of merit.

### Scraps and Gleanings.

Deaths from cholera in Jeddah number over 100 a-day.

Surgeon Parke is to be attached to the 2nd Life Guards.

Four women last week took their medical degree at Paris.

Two deaths in London last week were due to the excessive heat.

The Medical Academy for Women at St. Petersburg has re-opened.

It is proposed to enlarge Luton Cottage Hospital at a cost of about £450.

Lady Lyall is organizing a fancy fair in aid of Lady Dufferin's Fund.

About 70,000 persons die annually in the United Kingdom from consumption.

Pontefract Dispensary is going to build an accident ward, at a cost of about £100.

A lady has died at Lille from the effects of cocaine injected into her gums by a dentist.

Halstead Cottage Hospital issues a good report; there is a balance of £48 at the bank.

Miss Barr, of Cupar-Fife, has presented £10,000 to the Victoria Infir-mary for a new wing.

Two sacred concerts have been given at Leeds in aid of the Work-people's Hospital Fund.

Dr. Ord, of St. Thomas's Hospital, distributed the prizes to the successful Netley pupils.

The workmen of Saltley Gasworks have handed over £47 to the Hospital Saturday Fund.

The Cremation Conference have appointed a committee for the furtherance of cremation.

Hoyland, Mapplewell, and Staincross have held a Hospital Sunday in aid of the Barnsley Becket Hospital.

The death is announced, with deep regret, of Mr. W. B. Croker, house-physician to Belfast Royal Hospital.

A bazaar has been held at Scarborough in aid of the King's Cliff Hospital, in which Lady Sitwell is interested.

Laermanns has been sentenced to 15 years' penal servitude for causing the death of Mrs. Hall by an illegal operation.

Trowbridge Church Parade, in aid of the Bath and Warminster Hospitals, resulted in a collection of over £20.

The Medical Committee of Cheltenham General Hospital are against the reception of diphtheria cases into the wards.

Bingley Cottage Hospital was opened by Mr. Barran, M.P., on July 26th. The building has cost just under £2,000.

A convalescent Home has been opened at Witley, near Godalming. Charges from 7s. to 15s. a-week; women and girls only.

The Louisville Charity Commissioners have adopted a resolution that women in the workhouse should be made to break stones.

The Liquor Carnis Company, Holborn Viaduct, will send memoranda tablets to any medical practitioner who cares to apply for one.

Miss Skuers, Miss Sutherden, and Miss Jay, have taken the three first places at the late examination in botany at the Apothecaries Hall.

Dundee is getting frightened at the possible expense of the proposed Poor-law Hospital, and has decided not to decide on the question just yet.

Owing to the spread of the cholera in Spain, the Morocco Board of Health has re-established quarantine against arrivals from Gibraltar and Spanish ports.

The result of the first Hospital Saturday at Barnet is most gratifying to those concerned. Over £180 was collected, and the whole debt on the land has been paid off.

Ancoats Hospital, Manchester, has a deficit of £675. Nearly 11,000 persons were treated last year. The workpeople are to the fore in sup-porting this institution.

Owing to the growing danger from the pollution of the Ganges by the sewage of riparian towns, the Calcutta Health Society is addressing the Government on the subject.

Lewes Hospital charges each in-patient 10s. 6d. a-week. Alderman Kemp says he will be glad when their financial condition will permit them to abolish this charge.

M. Balma Olette has discovered a specific against cholera; you must sit for several hours in a shirt soaked in paraffin. Even Job's friends wouldn't have come near him under these circumstances!

Carlisle Hospital Sunday Fund has been divided as follows: Cumberland Infirmary, £500; Selloth Convalescent Institution, £100; and Carlisle Dispensary, Fever Hospital, and Workington Infirmary, £50 each.

The scandals at Croydon Infirmary have concluded with the dismissal of the steward. It is to be hoped the medical superintendent will exer-cise more watchful care over his subordinates in future, and get a better class of nurses.

At the closing fête of the International Medical Congress at Berlin on Saturday, the doctors of Berlin entertained their foreign visitors, and over 6,000 guests were present. The Empress officially received a number of the delegates.

The Warneford Hospital at Leamington is about to be altered and enlarged, from plans by Mr. Keith D. Young, of London. The works will include the provision of better sleeping accommodation, a recreation and dining room for the nurses, rooms for a junior house-surgeon, and more beds for the patients. The outlay will be between £8,000 and £9,000.

In view of the universal interest aroused by the Kemmler execution, an accident which occurred on Friday to an employé of an Electric Light Company at Washington has attracted great attention. By an inad-vertance, the man received a shock of 2,000 volts. He became insen-sible, but soon recovered, though the places where the current entered and left his body showed marks of burning, and were very painful.

